# Effect of glucose mediated oxidative stress on apoptotic gene expression in gingival mesenchymal stem cells

**DOI:** 10.1186/s12903-021-02007-y

**Published:** 2021-12-18

**Authors:** Rabiya Junaid, Mohsin Wahid, Farzeen S. Waseem, Rakhshinda Habib, Arshad Hasan

**Affiliations:** 1 Dow Research Institute of Biotechnology and Biomedical Sciences, Dow University of Health Sciences, DUHS, Karachi, Ojha Campus Pakistan; 2 Department of Oral Biology, Dr. Ishrat-Ul-Ebad Khan Institute of Oral Health Sciences, Dow University of Health Sciences, Karachi, Pakistan; 3 Department of Pathology, Dow International Medical College, Dow University of Health Sciences, Karachi, Pakistan; 4 Department of Operative Dentistry, Dow Dental College, Dow University of Health Sciences, Karachi, Pakistan

**Keywords:** Stem cells, Dental mesenchymal stem cells, Gingival mesenchymal stem cells, Oxidative stress, Hyperglycemia, Apoptotic gene expression, Growth kinetics

## Abstract

**Background:**

Diabetes is a common disease that causes gingival and periodontal problems. Stem cells isolated from dental sources are an emerging area of research with a potential to facilitate regenerative medicine. The stem cells retain the property of self-renewal and the ones isolated from dental sources are mainly multipotent mesenchymal stem cells that have the ability to self-renew as well as differentiation towards multiple lineages.

**Objectives:**

We aimed to isolate and characterize gingival mesenchymal stem cells by pluripotency markers and investigated the effect of oxidative stress on growth kinetics and apoptotic gene expression of gingival cells exposed to glucose mediated oxidative stress.

**Methods:**

In this study, we isolated gingival mesenchymal stem cells from gingiva. This was followed by morphologic analysis using inverted phase contrast microscopy and molecular profiling of these cells for the mRNA expression of specific genes. The isolated cells were cultured till passage 3 and then exposed to oxidative stress (high glucose concentration). We measured the apoptotic gene expression and compared their growth kinetics.

**Results:**

The results showed that oxidative stress produced by glucose reduced growth kinetics and increased apoptotic gene expression in gingival mesenchymal stem cells. According to the genetic results, glucose activated TNF family to initiate apoptosis.

**Conclusion:**

In conclusion, the present study demonstrated that high glucose obliterated cellular proliferation testified by evaluating growth kinetics and induced apoptotic gene expression in gingival mesenchymal stem cells. This initiated extrinsic apoptotic pathway mediated by TNF family. Therefore, in diabetes oral health condition is compromised and periodontal disease is common.

**Supplementary Information:**

The online version contains supplementary material available at 10.1186/s12903-021-02007-y.

## Background

The human body is comprised of millions of cells that are associated with distinctive functions in a differentiated state, whereas some cells remain in an undifferentiated dormant state. These immature cells become activated at the time of injury or repair as they have the ability to self-renew and are able to differentiate into one or more specialized cell types, thus they are called stem cells [1].

Mesenchymal stem cells have gained a lot of popularity as they can be obtained easily from adult tissues [2]. A range of stem cells can be obtained from the tooth that are resided in “stem cell niches” of their respective tissues and are referred to as dental mesenchymal stem cells (DMSCs). The dental sources include dental pulp stem cells (DPSCs), stem cells from deciduous teeth (SHED) both are obtained from pulp chambers of a permanent and primary tooth, stem cells from apical papilla (SCAP), dental follicle stem cells (DFSCs), Periodontal Ligament Stem Cells (PDLSCs) and Gingival Mesenchymal Stem Cells (GMSCs) that can be isolated from the lamina propria of gingiva [3, 4].

Although many dental mesenchymal stem cells are located in different parts of oral cavity, their drawbacks include availability of limited amount of tissue, elective endodontics and extraction is required to obtain the sample. Human gingival mesenchymal stem cells (GMSC) are considered as a better source because they do not require such procedures and can be isolated easily from human gingiva [5]. They are resided in gingiva in dormant state that on activation initiates healing and regeneration. The characteristic features include faster rate of proliferation, homogenous spindle shape morphology and stable features [6].

GMSC express CD73, CD44, STRO-1, CD105, CD90, and CD166, and are negative for hematopoietic markers CD34, CD45, and CD14. These cells also display multi-lineage capacity, which means they can be differentiated into adipogenic, osteogenic and chondrogenic cell lines [5]. Numerous studies have been published on GMSC that describe their adipogenic and osteogenic proliferative potential, molecular characterization and osteogenic reconstruction abilities [7–9]. Some studies demonstrate that these cells exhibit pluripotent markers OCT4, SOX2, Nanog and more [10], and have the ability to form neurogenic tissues [11].

Diabetes Mellitus is one of the most common metabolic disorders that interfere with the metabolism of proteins, carbohydrates and fats. Fibroblasts are the predominant cells in gingival and periodontal tissue and they are associated with collagen formation and degradation, and due to this reason wound healing depends on it. When the levels of glucose are raised in the body, oxidative stress is produced resulting in slower migration and proliferation of cells [12]. The gingival epithelial cells form the outer most covering of gingiva and they have shown cellular changes under diabetic conditions [13]. Moreover, their proliferation and migration is disrupted, inflammation is enhanced due to inflammasome and caspase 1 inhibition [14], reactive oxygen species are increased and phosphorylation of PI3K/Akt and MAPK/Erk signaling impaired resulting in compromised wound healing [15].

Oxidative stress is a dose-dependent condition and is produced because of overproduction of ROS. First, inflammation is initiated in its response and antioxidants are activated. The antioxidants halt the detrimental reactions and restore the normal cell function and morphology. If the inflammation persists for a long time, then it will produce pathological cascade leading to apoptosis of cell [16].

There are two main pathways in a cell that can trigger apoptotic cell death. In extrinsic pathway, the primary role is played by cell membrane receptors, the death receptors, which become activated when stimulated by surrounding cells, such as macrophages and natural killer cells. These receptors, named as TNFR1, TNRF2, DR1, DR2 and Fas, have the ability to initiate the apoptotic cascade by activating CASP8 and CASP 10 [17]. However, CASP8 may also down regulate the other initiator caspases in this pathway and may halt the progression of apoptotic stimuli [18].

Intrinsic apoptotic pathway is also called mitochondrial pathway that is triggered from DNA damage, cytotoxic drugs, metabolic stress and lack of blood supply. It is mainly regulated by BCL2 proteins and the genes BAX and BAK increase permeability of outer mitochondrial membrane (MOMP) that results in the secretion of cytochrome c in the cytoplasm [19]. This cytochrome c induces conformational change in APAF1 that in turn activates CASP 9 [18], and it further activates executioner proteins like CASP 3, 6 and 7 [20].

Understanding the behavior of GMSCs is extremely crucial because they are subjected to oxidative stress numerous times in a day. Their location in the oral cavity increases their susceptibility, as they have to frequently bear mechanical injuries and variations of temperatures. Likewise, these cells also have to tolerate the fluctuations of glucose, blood flow and bacterial or fungal infections. These all stresses on cells increase apoptosis and effect repair and regenerative capabilities of cells.

In this study, we aimed to find out the most suitable culturing conditions for gingival mesenchymal stem cells and mimicked hyperglycemic environment. The behavior, growth kinetics and apoptotic gene expression of control and hyperglycemic treated GMSC was compared. Although previous studies showed that growth of GMSCs is effected and ROS production is increased in hyperglycemia, the expression of apoptotic genes is not well explored under such kind of oxidative stress.

## Materials and methods

### Sample collection

In this study, three samples of healthy gingival tissue were collected from donors who provided informed consent. These patients were undergoing surgical procedures, such as third molar extraction and crown lengthening in the Oral Surgery Department of Dow University of Health Sciences (DUHS). The patients with coronal caries, pulpitis, apical periodontitis, fractured and necrosed teeth were excluded. The study was approved by Institutional Review Board and Board of Advanced Studies & Research of DUHS. A small section of gingival tissue (2 × 1 × 1 mm) was excised after the administration of local anesthesia. The excised gingival tissue was transported to Dow Research Laboratory (DRIBBS) for further processing in PBS at 4 °C to retain the vitality of tissue.

### Isolation of gingival mesenchymal stem cells

This procedure has been accomplished by outgrowth/explant method in which excised gingival tissue was placed on petri dish and washed with PBS. The gingival tissue was minced in petri dish and then the pieces of tissue were centrifuged at the speed of 1300 rpm in room temperature for 5 min. The supernatant was discarded and the pellet was re-suspended in complete media. It was placed in a well of 6 well plate with 1500 µl of complete culture media that contained Dulbecco’s modified eagle’s medium (DMEM) with 10% fetal bovine serum (FBS) and 1% Antibiotic–Antimycotic. The culture medium was changed after every third day and they were trypsinized when 70%-80% confluency was reached.

### Total RNA extraction & real time PCR

The RNA was extracted by following Allprep DNA/RNA kit (Qiagen, cat no. 80204) protocol and 30 µl of elution volume was obtained. To evaluate quantity of RNA, Nanodrop Lite Spectrophotometer (cat no. ND-LITE, Thermo Fisher Scientific) was used and integrity was checked by Agilent 2100 Bioanalyser (Model G2939B). TaqMan MicroRNA Reverse Transcription Kit (cat no. 4366596, Applied Biosystems, Thermo Fisher Scientific) was used to synthesize cDNA. For qPCR reactions, the expression of OCT4, SOX2, Lin28 and 18s rRNA (the housekeeping gene) was detected through Quant Studio™ 7 Flex Real-Time PCR System (Applied Biosystems) along with TaqMan Fast Advanced Master Mix (cat no. 4444557, Applied Biosystems, Thermo Fisher Scientific). In this experiment, 18s rRNA was taken as control and the expression level of other genes was normalized with it.

### Culturing under oxidative stress & morphological analysis

The morphological changes were seen in the cells under inverted phase contrast microscope (Leica Dmi1; Leica Microsystems CMS GmbH) and images were captured simultaneously. Sterile solution of glucose (Gibco, cat no. A24940-01) was added in the culture media to prepare 75 mM concentration of glucose, and this was transferred to T25 flask and left for 72 h. Later on, RNA was isolated and apoptotic genes were checked by running apoptotic gene array card.

### Growth kinetics

When the cells became 70–80% confluent, the viable cell count taken from Vi-Cell Counter (Beckman Coulter Vi-Cell XR) were plated in 6 well plate each well containing 60,000 cells/ plate. The reaction was done in triplicates and two doses of glucose 50 mM and 75 mM were tested with the control group for 72 h. The dilution factor was taken as 10 and the analysis was done in a Vi-Cell XR. It produced viability readings and cell count of each concentration, which we compared with the control reading. To simplify experimental groups and to replicate the hyperglycemic environment solely the 75 mM glucose concentration was used in the following experiments.

### Statistical analysis

Statistical test one-way Anova and Tukey post hoc test was applied for comparison of groups. This test is used to compare the mean of groups that have been split in two independent variables. It explains the interaction between two independent variables on the dependent variables. The version of SPSS software was 16.0 and the *p* value < 0.05 was considered statistically significant. To analyze fold change of apoptotic genes the software Graphpad prism version 9.2 was used and *p* value was taken as above.

### Gene expression analysis by quantitative real time PCR

At passage 3, both the treated and untreated cells (control) were selected for gene expression analysis involved in Human Apoptosis using TaqMan Array Human Apoptosis Panel (cat no. 4378716, Applied Biosystems). Each card contained 8 ports/ reservoirs for filling, and 30 ng of RNA was reverse transcribed to cDNA to fill each reservoir. The micropipette was used to load the 100 µl of PCR mixture and then the tip was placed on fill port of card in an angled position. The accurately filled card was sealed, centrifuged and placed in qPCR. The human apoptotic assay card was run in Quant StudioTM 7 Flex Real-Time PCR system (Applied Biosystems). The setup file was imported in the PCR software while TaqMan probes and fast cycling method was opted for fast advanced master mix reaction.

#### 2-delta delta CT (2^−∆∆CT^) method

To quantify the genetic expression of genes 2 −∆∆CT approach is commonly and widely used as it is a relative quantification method. In this technique, we calculate the relative gene expression level of untreated (control) and treated (target) samples. The CT values are acquired from qPCR and reference or housekeeping gene is used to normalize the genetic data. The threshold cycle (CT) is defined as the number of cycles that are needed for the level of fluorescence to reach a certain amount or threshold. In qPCR positive result is perceived by the assembling of fluorescence.

In this study, we have used three housekeeping genes that are 18s rRNA, GAPDH and Actin Beta to normalize the data. The calculation is done as follows:

- First, subtract the CT level of reference gene from target gene to get ∆CT value.
- Second, subtract the ∆CT of reference sample from target sample and the result shows the value of ∆∆CT
- Finally, the result of (2^−∆∆CT^) is expressed as fold change in genetic expression of target genes.

The CT values have an inverse relation with the quantity of target gene in sample, so lower values denotes high expression while higher levels denotes low genetic expression.

## Result

### Primary culture and morphologic assessment of GMSC

**Fig. 1:**
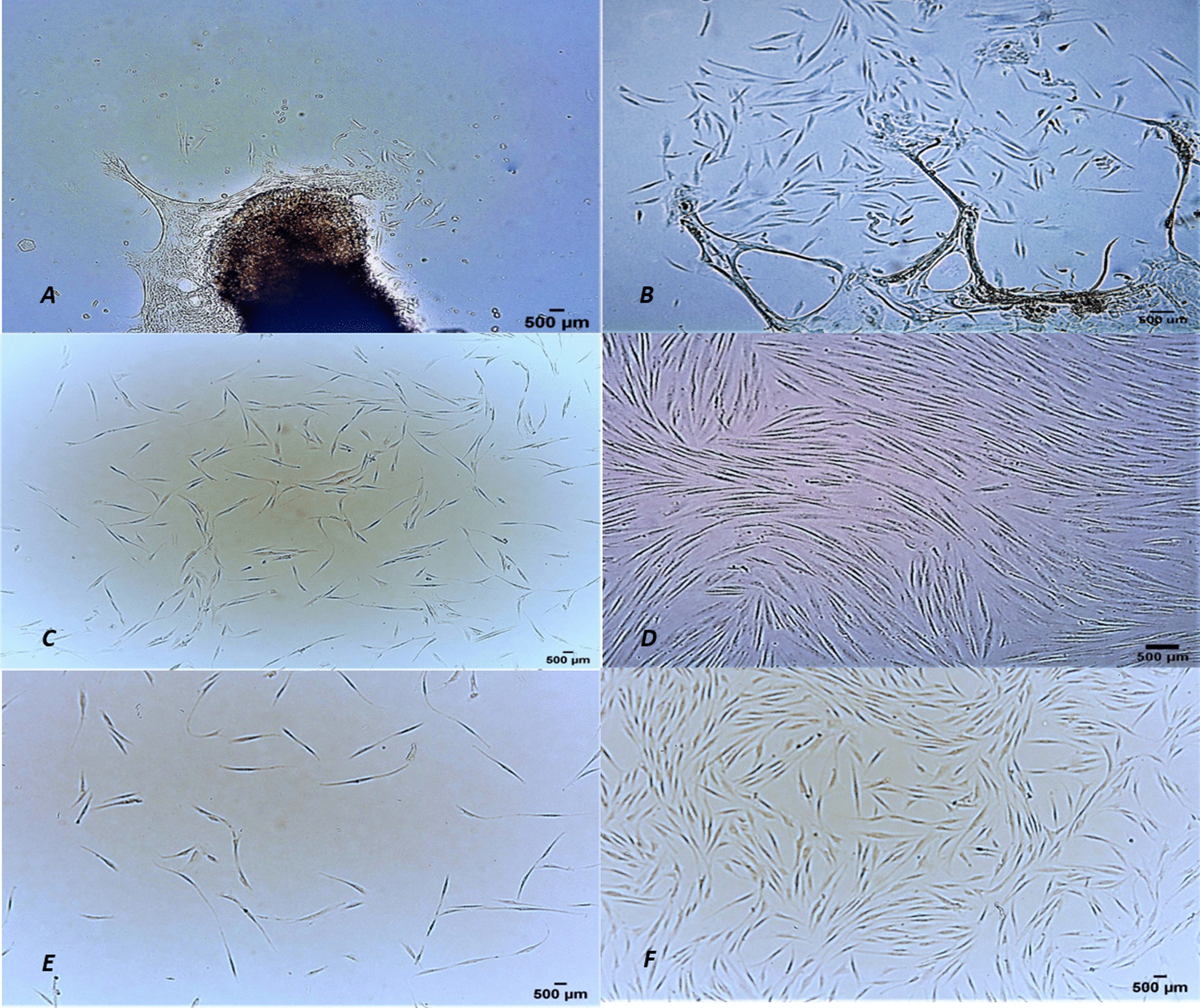
The figure shows spindle shape morphology of gingival mesenchymal stem cells and their proliferation. **A** Day1—The cells first appeared on 8th day of isolation. **B** Cell numbers increased due to proliferation on day 4. **C** On day 8, the non-adherent cells were removed with each wash. **D** Cells reached confluency on day 12 of their first appearance. **E** It shows initial attachment of cells after 24 h of passage. **F** GMSC 70–80% confluent after passage. (Scale bar = 500 µm)

After isolating cells by outgrowth method, the first cluster of cells appeared from the tissue in between 8 and 12 days. Under phase contrast microscope, the cells were spindle shaped that had wider-oval shaped cell body with bulbous nucleus and elongated edges. The monolayer appeared at the bottom of plate and the cells that did not adhere were removed with each wash. The microscopic and first appearance of cells can be seen in the figure below (Fig. 1).

### Integrity of RNA

It is important to check the integrity of RNA for the succeeding Real-time PCR reactions because degraded RNA produce variation in gene expression. The bioanalyser software generates ribosomal ratios (18 to 28s) that show the intactness of RNA because the degradation of RNA occurs gradually and results in decreasing the ratio. Therefore, the results of bioanalyser are reproducible and reliable as the human error is reduced. In the current study, the RNA of control and treated GMSC were investigated, which produced proper peaks on the electropherogram of bioanalyser.

### Characterization of GMSC by mRNA expression

The real-time PCR provides the advantage of assessing amplified product with completion of each PCR cycle and the amplification of each DNA template is directly proportional with the fluorescence signal.

**Fig. 2:**
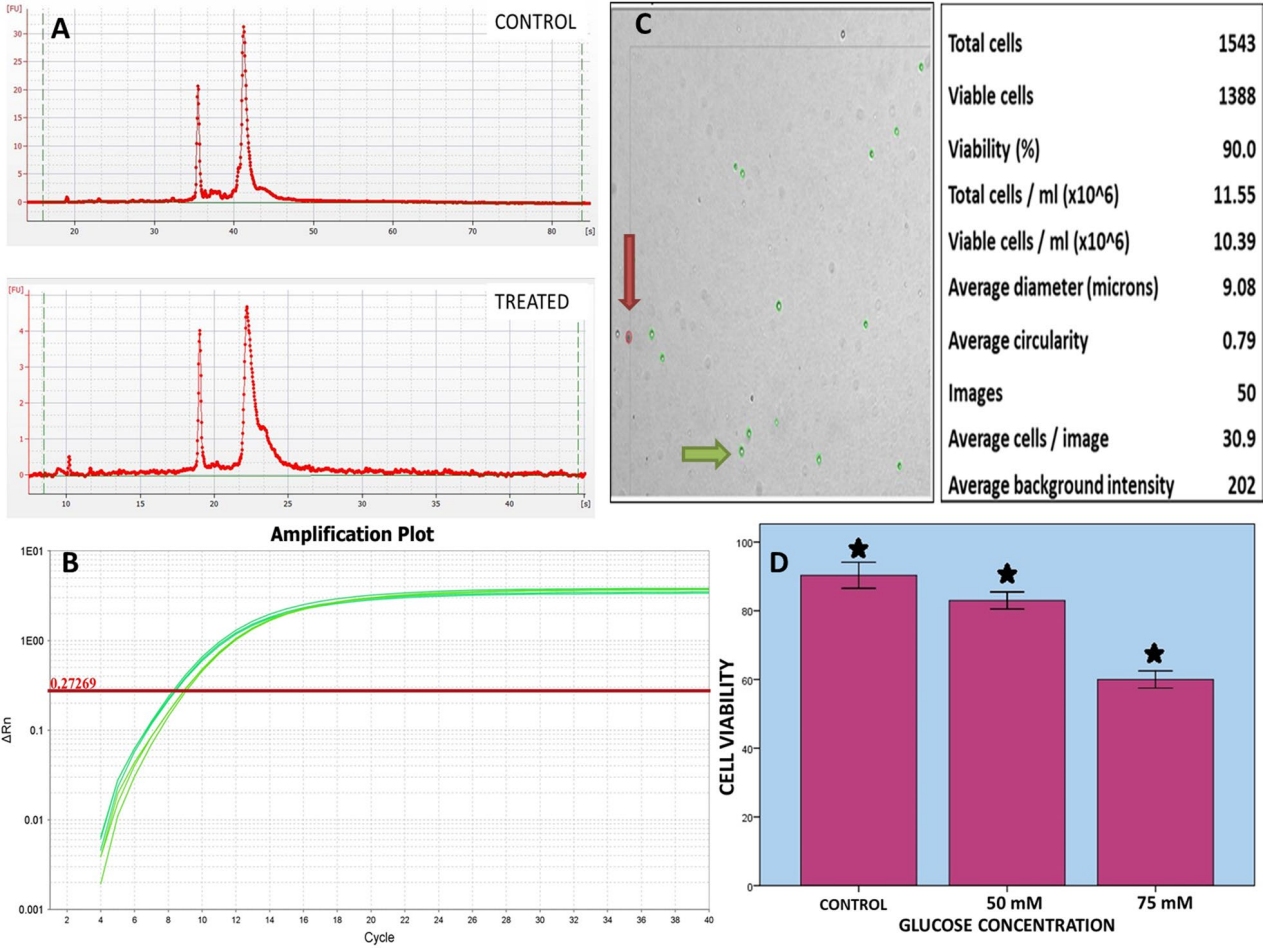
**A** The integrity of RNA extracted from GMSC was analyzed before and after glucose treatment. The two sharp peaks at 18s and 28s ribosomal subunit on electropherogram depicted good quality of RNA. **B** The house keeping gene-18s was examined between control and treated samples in triplicates. The mean CT value of control was 8.896 and mean CT value of treated sample was 8.310. **C** The viable GMSC are shown with green arrow and non-viable are shown with red arrow. **D** The figure shows the effect of glucose on GMSC viability and each bar represents the mean ± SD values of three independent experiment (*P < 0.05 as compared to control)

In our study, the qPCR or real time PCR was used for characterization of GMSC using pre-formed primers of 18s (the housekeeping gene), Lin28, SOX2 and OCT4. The cells showed the positive expression of these stemness related markers (Table 1; Fig. 2).

**Table 1:** CT mean values of the genetic markers of study compared with HK gene

GENE MARKER	CT VALUE (MEAN)
*OCT4*	31.272
*SOX2*	32.276
*Lin28*	32.775
*18s*	8.326

### Growth kinetics under glucose mediated oxidative stress

To examine the effect of glucose mediated oxidative stress on GMSC, cell viability was determined using Vi-Cell Counter XR. The cells were plated in 6 well plate in equal numbers and viability at control was compared with glucose at two concentrations, 50 mM and 75 mM, after exact 72 h. The percentage of viable cells in control was around 90–92%, whereas at 50 mM concentration not a significant reduction in viability was seen. Despite this, the third concentration 75 mM showed absolute reduction in viability percentage of cells and the results were statistically significant.

### Expression of molecular mediators involved in regulation of apoptosis

The GMSC at P3 were left in hyperglycemic media with 75 mM concentration of glucose for 72 h. Then the RNA was extracted from treated cells and low concentration of RNA was obtained due to the decreased confluency of cells. The reading after spectrophotometry was 49.1 ng/µl and A260nm/A280nm ratio was 2.10. To proceed further, cDNA synthesis of treated cells was accomplished and Human Apoptosis microfluidic card was run on qPCR. The gene expression data was normalized with three housekeeping gene and then fold change was observed (Table 2) (Additional file 1).

**Table 2:** Average of 2 (-ΔΔCT) Values in Apoptotic Genes Depicted Fold Change

Target Name	18S (2^-∆∆CT)	ACTB (2^-∆∆CT)	GAPDH (2^-∆∆CT)	Average of (2^-∆∆CT)	Target Name	18S (2^-∆∆CT)	ACTB (2^-∆∆CT)	GAPDH (2^-∆∆CT)	Average of (2^-∆∆CT)
APAF1	1.28	1.72	0.81	1.27	BAX	1.43	1.92	0.90	1.42
BAD	0.65	0.87	0.41	0.64	BBC3	0.92	1.24	0.58	0.91
BAK1	0.92	1.24	0.58	0.92	BCAP31	0.97	1.31	0.62	0.97
BCL10	1.93	2.59	1.22	1.91	DEDD2	1.24	1.66	0.78	1.23
BCL2A1	4.80	6.46	3.04	4.76	DEDD	2.50	3.36	1.58	2.48
BCL2	0.09	0.12	0.06	0.09	DIABLO	1.39	1.87	0.88	1.38
BCL2L10	0.95	1.28	0.60	0.95	ESRRBL1	1.13	1.52	0.72	1.12
BCL2L11	3.49	4.70	2.21	3.46	FADD	0.83	1.12	0.52	0.82
BCL2L13	1.53	2.06	0.97	1.52	FAS	1.19	1.60	0.75	1.18
BCL2L14	29.80	40.13	18.86	29.59	FASLG	18.53	24.95	11.73	18.40
BCL2L1	1.36	1.83	0.86	1.35	HIP1	1.59	2.14	1.01	1.58
BCL2L2	1.32	1.78	0.83	1.31	HRK	0.59	0.80	0.37	0.59
BCL3	2.40	3.24	1.52	2.39	HTRA2	2.04	2.75	1.29	2.02
BID	0.96	1.29	0.61	0.95	ICEBERG	0.04	0.05	0.02	0.04
BIK	0.95	1.28	0.60	0.95	IKBKB	1.43	1.93	0.91	1.42
BIRC1	0.79	1.06	0.50	0.78	IKBKE	2.70	3.63	1.71	2.68
BIRC2	2.05	2.76	1.30	2.04	IKBKG	1.21	1.63	0.77	1.20
BIRC3	0.80	1.08	0.51	0.79	LRDD	1.51	2.04	0.96	1.50
BIRC4	1.82	2.46	1.15	1.81	LTA	4.23	5.69	2.67	4.20
BIRC5	0.36	0.48	0.23	0.35	LTB	0.58	0.78	0.37	0.58
BIRC6	1.98	2.67	1.25	1.97	MCL1	1.43	1.93	0.91	1.42
BIRC7	0.95	1.28	0.60	0.95	NALP1	2.18	2.93	1.38	2.16
BIRC8	0.67	0.90	0.42	0.66	NFKB1	1.94	2.61	1.23	1.92
BNIP3	1.66	2.24	1.05	1.65	NFKB2	1.69	2.27	1.07	1.68
BNIP3L	2.53	3.41	1.60	2.51	NFKBIA	4.68	6.30	2.96	4.64
BOK	0.89	1.20	0.56	0.89	NFKBIB	1.56	2.09	0.98	1.54
CARD15	1.68	2.27	1.07	1.67	NFKBIE	1.41	1.90	0.89	1.40
CARD4	0.90	1.21	0.57	0.89	NFKBIZ	0.72	0.97	0.45	0.71
CARD6	3.48	4.69	2.21	3.46	PEA15	1.53	2.05	0.97	1.52
CARD9	0.24	0.33	0.15	0.24	PMAIP1	5.43	7.31	3.44	5.39
CASP10	0.72	0.97	0.46	0.71	PYCARD	1.87	2.51	1.18	1.85
CASP14	0.95	1.28	0.60	0.95	RELA	1.91	2.58	1.21	1.90
CASP1	1.36	1.83	0.86	1.35	RELB	0.91	1.22	0.57	0.90
CASP2	1.56	2.10	0.99	1.55	REL	2.24	3.02	1.42	2.23
CASP3	1.91	2.58	1.21	1.90	RIPK1	2.81	3.78	1.78	2.79
CASP4	1.59	2.14	1.01	1.58	RIPK2	1.99	2.68	1.26	1.98
CASP5	1.37	1.85	0.87	1.36	TA-NFKBH	1.37	1.85	0.87	1.36
CASP6	1.70	2.29	1.08	1.69	TBK1	2.97	3.99	1.88	2.95
CASP7	1.74	2.34	1.10	1.73	TNF	99.15	133.52	62.75	98.47
CASP8AP2	2.06	2.77	1.30	2.04	TNFRSF10A	1.84	2.48	1.17	1.83
CASP8	2.11	2.84	1.34	2.10	TNFRSF10B	1.15	1.55	0.73	1.15
CASP9	1.00	1.34	0.63	0.99	TNFRSF1A	1.79	2.41	1.13	1.78
CFLAR	1.79	2.40	1.13	1.77	TNFRSF1B	1.87	2.52	1.19	1.86
CHUK	1.60	2.16	1.01	1.59	TNFRSF21	0.56	0.75	0.35	0.55
CRADD	1.15	1.55	0.73	1.15	TNFRSF25	0.85	1.15	0.54	0.85
DAPK1	3.29	4.43	2.08	3.26	TNFSF10	46.77	62.98	29.60	46.45

### Glucose effects on gene expression of apoptotic genes

**Fig. 3:**
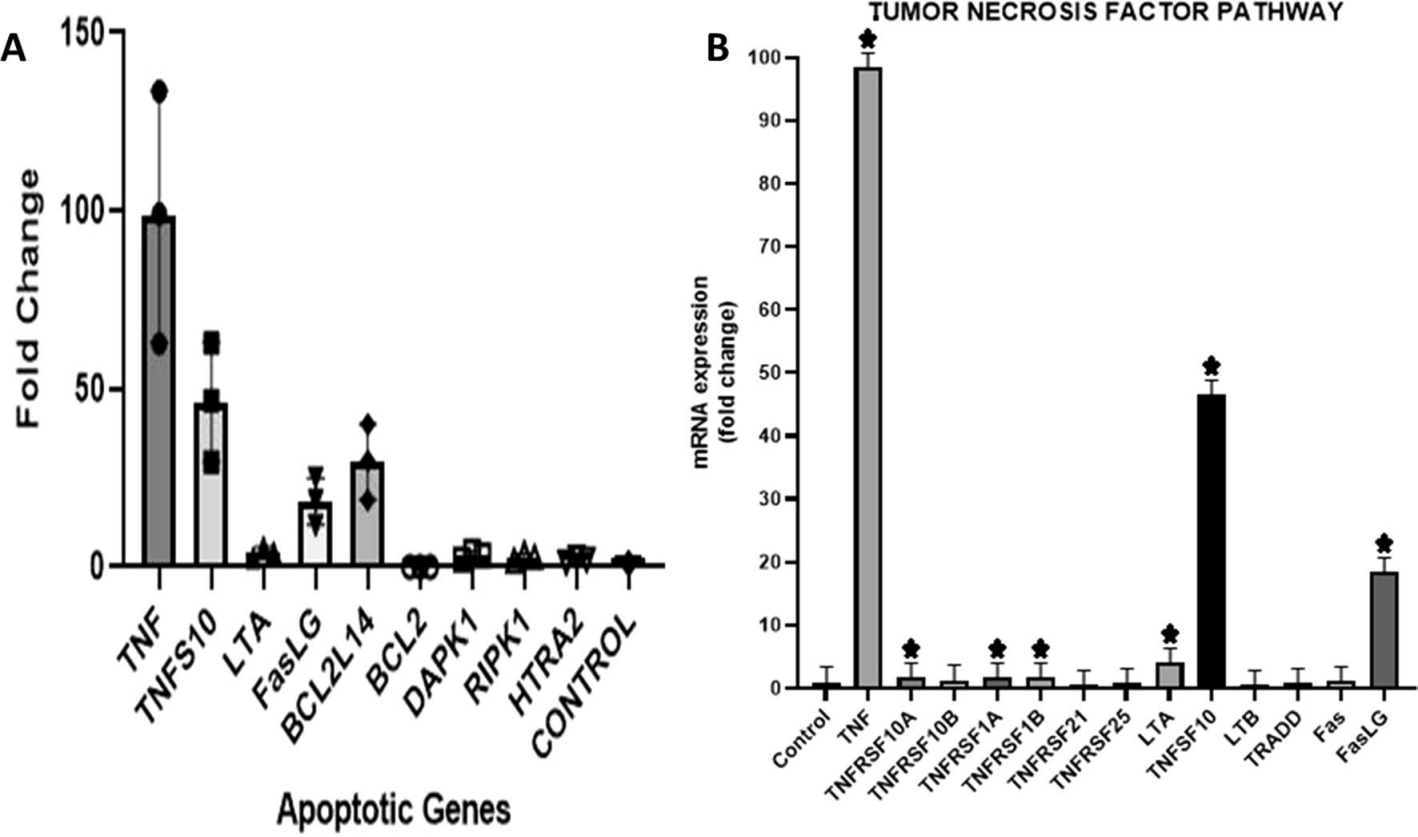
The data has been normalized by 3 housekeeping genes that are 18s, GAPDH and ACTB. **A** The figure includes apoptotic genes that show significant fold change. They mainly belong to TNF family and follow extrinsic apoptotic pathway for apoptosis. The statistical analysis was carried out using One-way ANOVA and post hoc tukey test that showed *P* value less than 0.05. **B** In this graph marked fold change can be appreciated in TNF, TNFSF10, TNFSF10A, LTA, TNFRSF1A and Fas LG against control (equal to 1). The other remaining genes were also increased (The fold change was considered significant if its value was above or around 2)

**Fig. 4:**
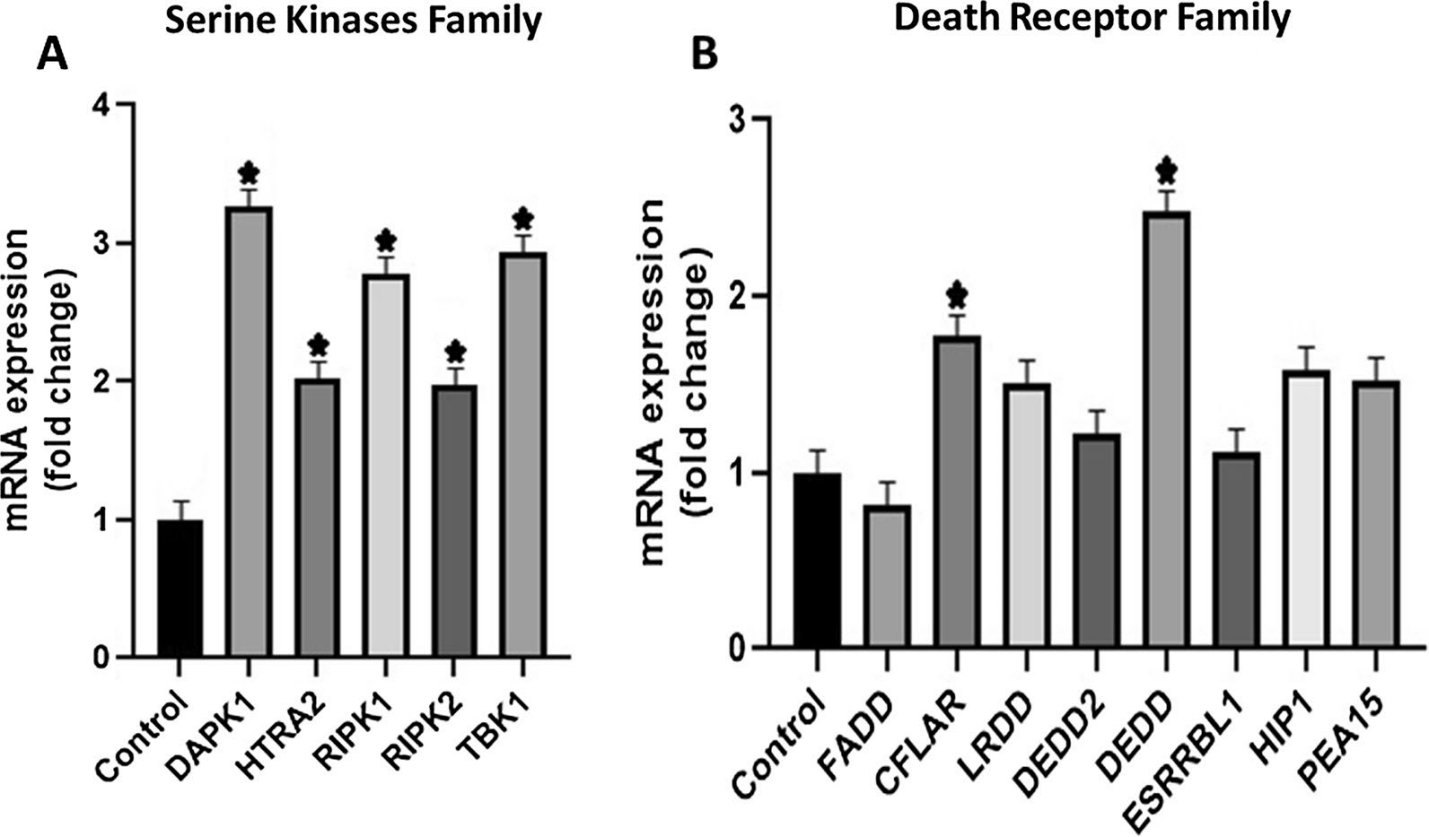
**A** The genes DAPK1, HTRA2, RIPK1 and TBK1 are related to serine kinases family show increased mRNA expression. **B** The gene DEDD and CFLAR has shown significant fold change. (*P value ≤ 0.05)

The mRNA expression levels of apoptosis genes were studied by qPCR. The genes of BCL2 family were divided into pro-apoptotic genes and anti-apoptotic genes**.** The control was taken as 1 and the values at or around 2 were regarded as fold change. The glucose was shown to initiate apoptosis in gingival mesenchymal stem cells (GMSC) through extrinsic TNF pathway as studied by the outcomes of Real-time PCR. The outcome revealed statistically significant up-regulation of TNF, FasLG and TNFSF10 gene and others related to this pathway (Figs. 3, 4).

**Fig. 5:**
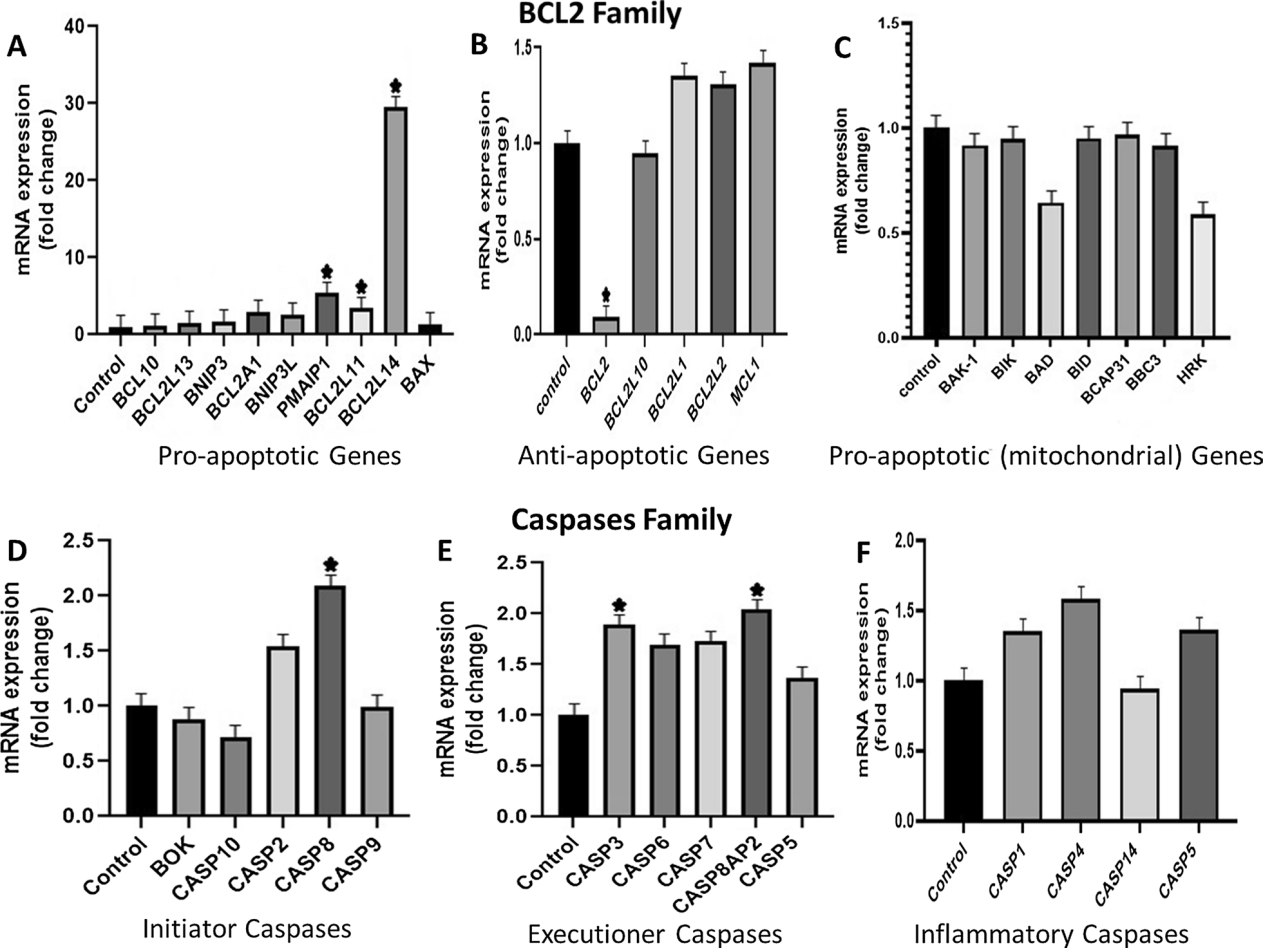
**A** This graph shows pro-apoptotic genes of BCL2 Family. The genes BCL2L14, PMAIP1 and BCL2L11 showed significant fold change. **B** Anti-apoptotic genes of BCL-2 family were also down-regulated, especially BCL2 gene. **C** It shows down regulated pro-apoptotic genes of BCL-2 family that mainly play role in mitochondrial apoptosis. **D** This figure includes initiator caspases in which only Casp 8 was increased because it is primarily involved in extrinsic pathway of apoptosis. **E** The executioner caspases displayed lower expression, except CASP8AP2 gene. **F** All inflammatory caspases show reduced expression. However, the difference among most genes was not found to be statistically significant. (*P value ≤ 0.05)

The family of serine kinases belongs to molecules of intermediary group that recruit molecules to activate apoptotic genes and the noticeable increased mRNA expression of DAPK1, RIPK1 and TBK1 was seen. BCL2 anti-apoptotic gene showed significant down regulation, whereas BCL2L14, PMAIP1 and BCL2L11 pro-apoptotic genes showed marked up-regulated expression. The gene BCL2L14 is known as apoptosis facilitator and PMAIP1 inhibits anti-apoptosis genes. Despite this, other genes of BCL2 family did not show any significant difference in the fold-change (Fig. 5).

**Fig. 6:**
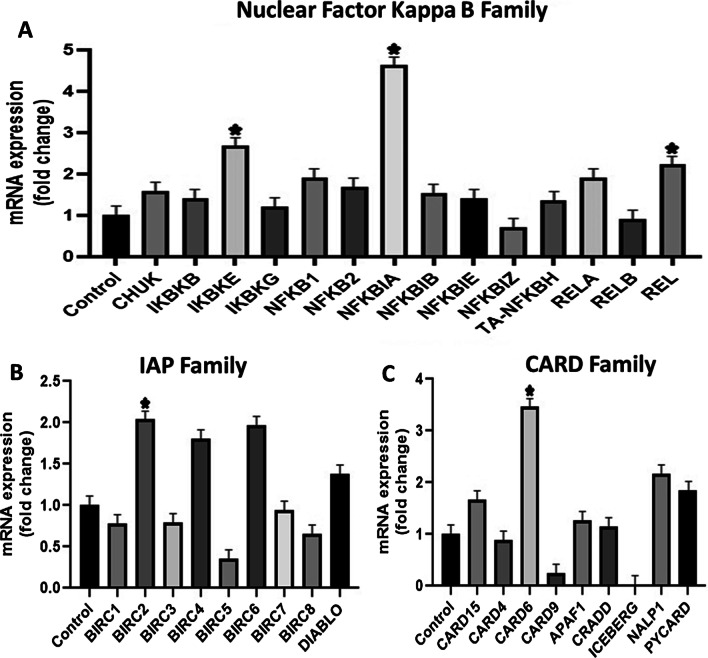
The figure shows fold change in different apoptotic families relative to the control = 1. **A** In NF-KB pathway, the gene expression of IKBKE, NFKBIA and REL was increased. **B** mRNA expression of IAP family did not show significant change except BIRC2. **C** This graph shows marked upregulated expression of CARD6, while the gene ICEBERG showed no expression. However, the difference among most genes was not found to be statistically significant. (*P value ≤ 0.05)

In caspase family, initiator caspase-CASP8 and executioner caspase-CASP8AP2 and CASP3 resulted in up-regulation, which is directly related to extrinsic pathway of apoptosis. Although most genes of Nuclear Factor Kappa B family displayed down regulated expression, the inhibitors of NFKB pathway—IKBKE and NFKBIA—showed increased expression. The gene REL was also amplified that form dimer combinations with other NFKB molecules when they are activated, therefore, it did not produce any effect (Fig. 6).

The CARD6 member of CARD family was markedly up-regulated that has the ability to activate CASP8 and other caspases, and subsequently, enhance extrinsically mediated apoptosis. The genes of IAP family have pro- and anti-apoptotic functions but significant change was not seen in this study except BIRC2 which regulates caspases and apoptosis.

## Discussion

Many oral health problems are related to Diabetes mellitus that adversely impacts overall quality of life. This happens because diabetic patients show gingival recession, bone loss, periodontal lesions and early tooth loss leading to compromised eating. Furthermore, the salivary glands are also affected causing xerostomia and burning mouth syndrome. The dental treatment options are often expensive and affordability might be a problem. Thus, the most common issue is periodontitis that is regarded as sixth diabetic complication [21].

The present study was an in vitro model that isolated and investigated the effect of hyperglycemia on stem cells of gingiva and related it to growth kinetics and apoptotic gene expression. The cell viability of GMSC was inspected under high and normal glucose level and then its genetic expression for apoptosis was explored.

The samples were taken from healthy individuals and the isolated cells appeared between 8 to 12 days from all the samples. The cells were isolated successfully through explant method and they showed characteristic features like spindle shaped morphology under phase contrast microscope and this was consistent with previously reported studies [6, 22]. To prove that these cells are gingival mesenchymal stem cells we selected markers, such as Lin28, SOX2, OCT4 and 18srRNA and they showed their expression, which was also published in a previous study. Gugliandolo et al. highlighted differential gene expression in GMSC cultured in plastic flasks and GMSC grown with a poly-lactic acid scaffold. The results of next generation sequencing showed up-regulation of survival and neurogenic genes and also revealed the contribution of P13K/Akt pathway, while the genes involved in apoptosis and growth inhibition of neurons were decreased [23].

To evaluate the difference in growth, the growth kinetics was investigated at hyperglycemic and normoglycemic concentration and GMSC showed apoptosis at increased concentration of glucose 75 mM although in lower and normal condition cells continued to grow at a faster pace. The inflammatory conditions in gingiva also increases apoptosis of cells, so any condition that disrupts the normal hemostasis of gingiva can result in decreased viability of cells [24]. A comparative study, analyzed growth curves for consecutive seven days in dental mesenchymal stem cells (including GMSC, DPSC, PDLSC and DFSC), umbilical cord mesenchymal stem cells, bone marrow mesenchymal stem cells and adipose tissue stem cells. They counted cells through a kit and reported that mesenchymal stem cells entered log phase of growth in first and second day. The proliferation rate of dental mesenchymal stem cells and umbilical cord stem cells was comparable and faster than other MSCs [25].

Our study showed that in higher glucose concentrations, oxidative stress increased due to ROS accumulation and ultimately led to apoptotic cell death in gingival mesenchymal stem cells. This hyperglycemic effect was testified in other earlier researches and in one of these studies hyperglycemic oxidative stress was induced to retinal pericytes of rat that resulted in increased ROS productions and apoptosis [26]. Another study had seen the same high glucose effect in human gingival fibroblasts and it reported that these cells showed impaired growth and migration of cells due to ROS accumulation [12].

We used low density array apoptotic array human panel in this study which contained 96 genes related to different apoptotic pathways. Some of the pro- apoptotic genes exhibited up regulation, while others were down regulated under high glucose concentration. In BCL2 family, the pro-apoptotic mRNAs that displayed more expression were BCL2L14, PMAIP1 and BCL2L11. The genes fundamentally involved in intrinsic pathway or mitochondrial pathway of apoptosis like BAX, BAK-1, BID, BAD, BIK, BCAP31, BBC3 and HRK were decreased and all anti-apoptotic genes especially BCL2 showed down regulation.

These results were consistent with a study that investigated hyperglycemic effects in pancreatic beta cells of mice, which revealed increased expression of Bbc3, Nix (BNIP3) and Bcl2l14 by real time PCR [27]. BCL2L14 markedly increased in our project is regarded as facilitator of apoptosis [28] and PMAIP1 is a pro-apoptotic gene and functions to neutralize anti-apoptotic activity of MCL1 and BCL2A1. This explains the down-regulation of anti-apoptotic gene activity in our result [29]. Apoptosis evaluated on cell line of oral squamous cell carcinoma claimed higher expression of BAX gene and p53 gene but the level of BCL2 anti-apoptosis gene was suppressed resembling our data [30].

Although apoptosis is a physiological process that helps to maintain the normal functions of tissues by confiscating non-operational cells, it also becomes active in pathological conditions e.g. Diabetes, kidney diseases. Over past few decades, apoptosis has received significant attention because keen interest is to minimize its effects by manipulating and blocking its causative genetic factors that might introduce novel treatment options for diabetic nephropathy [31].

In various pathological conditions, caspases are up regulated and can induce apoptosis by both extrinsic and intrinsic pathway. The relation of apoptosis and high glucose level were demonstrated with the help of qPCR in the current study. The results on GMSC revealed that higher expression of CASP 2 and 8 (the initiator caspases) was seen, while all the executioner caspases were increased including CASP3, CASP6, CASP7, CASP8AP2 and CASP5. These findings are consistent with the study reported by Chen et al. who showed higher Bax, Casp 9 and 3 levels and worked on mouse kidney mesangial cells [32].

In the present study, the TNF family and hyperglycemia was correlated and the genetic expression of TNF was maximized in GMSC and the same results have been seen in macrophages in 2020 [33]. Our study showed significant up regulation of TNF family members, such as TNFRSF1A, TNFSF10, LTA and more. Likewise, hyperglycemia in periodontal ligament stem cells showed impaired cellular functions and higher expression of TNFRSF1A (TNFR1). The reason behind its up-regulation was decreased formation and action of DNA methyltransferase that was an enzyme associated with methylation of the receptor-TNFRSF1A. Therefore, the viability of cells is highly decreased when this gene is hypomethylated and results in apoptosis of cells [34].

The gingival mesenchymal stem cells and periodontal ligament stem cells are the part of periodontium and both help in its regeneration and repair. Another study on PDLSC revealed similar results to the current work that greater amount of ROS was generated in high glucose environment and elevated levels of TNFRSF1A were displayed with increased apoptosis and decreased cell growth [35].

The genes of serine kinases are involved in several cellular pathways and they function as intermediary molecules. In a previous study, neuronal cells of mice were cultured and the apoptotic effect of DAPK1 was seen. An oxygen–glucose deprivation model was constructed which elaborated that during ischemia BCL2 genes as well as CASP3 was activated to induce apoptosis. They knocked down the effect of DAPK1 through lentiviral molecules and concluded that the apoptosis was reduced in neurons. Thus, DAPK1 and other serine kinases activated other crucial apoptotic pathways and in the present study as well its expression was increased [36].

Another member of this family RIPK1 that was up regulated in our study has shown to activate caspases and NF-KB apoptotic pathways. Once the receptor of TNF family-TNFRSF1A triggered, in turn activated RIPK1, which formed a complex with TRADD or FADD. This intermediate complex had the ability to induce apoptosis through CASP 8. On the other hand, if RIPK1 got ubiquitinated then it would activate nuclear factor kappa B pathway for apoptosis. Geng et al. had researched that RIPK1 molecule can alternatively activate mechanisms of cell death by the help of TAK1. TAK1 regulated the phosphorylation of RIPK1 and decided that whether RIPK1 would interact with FADD or RIPK3 to form a necrosome. TNF alone can directly activate CASP8 apoptosis this correlated with the lower expression of FADD and TRADD in our study [37].

The nuclear factor kappa B family was also investigated in our project and a study held in 2018 and they concluded that high sugar level in hepatic cells induces NF-κB pathway [38]. One more study on hyperglycemic concentration associated the increased levels of TNFR1 and TNFR2 with NF-κB in human endometrial cells. It stated that when TNF-α was amplified, the insulin effect was diminished causing intensification of hyperglycemia, and thereby augmented the impact of NF-κB proteins [39]. In hepatic cells, high glucose produced oxidative stress due to ROS generation which resulted in increased TNF-α and NF-κB proteins and induced apoptosis [40].

Finally, we evaluated the influence of hyperglycemia on apoptosis and growth kinetics of gingival mesenchymal stem cells. Our results revealed that the marked increased expression of TNF family with Fas L because of hyperglycemia in GMSC activated apoptosis through extrinsic apoptotic pathway. Moreover, apoptotic gene expression in GMSC is novel and the expression of some genes is not seen as per our knowledge.

## Conclusion

In conclusion, the present study demonstrated that high glucose obliterated cellular proliferation testified by evaluating growth kinetics and induced apoptotic gene expression in gingival mesenchymal stem cells. This was examined through human array apoptotic card run in Real-time PCR and hyperglycemia initiated extrinsic apoptotic pathway mediated by TNF family. Therefore, in diabetes oral health condition is compromised and periodontal disease is common.

## Supplementary Information

**Additional file 1**. **Table 1.** CT values of housekeeping genes. **Table 2.** The values of average CT for apoptotic genes. **Table 3.** Normalization of data with 18s housekeeping gene. **Table 4.** Normalization of data with ACTB and GAPDH housekeeping gene.

## Data Availability

The datasets used and/or analyzed during the current study are available from the corresponding author on reasonable request.

## References

[1] Kitadate Y, Jörg DJ, Tokue M, Maruyama A, Ichikawa R, Tsuchiya S, et al. Competition for mitogens regulates spermatogenic stem cell homeostasis in an open niche. Cell Stem Cell. 2019;24(1):79–92. e6. 10.1016/j.stem.2018.11.013 PMC6327111 30581080

[2] Tang L, Jiang Y, Zhu M, Chen L, Zhou X, Zhou C (2020). Clinical study using mesenchymal stem cells for the treatment of patients with severe COVID-19. Front Med.

[3] Granz CL, Gorji A (2020). Dental stem cells: the role of biomaterials and scaffolds in developing novel therapeutic strategies. World J Stem Cells.

[4] Venkatesh D, Kumar KM, Alur JB (2017). Gingival mesenchymal stem cells. Journal of oral and maxillofacial pathology: JOMFP.

[5] Du L, Yang P, Ge S (2016). Isolation and characterization of human gingiva-derived mesenchymal stem cells using limiting dilution method. J Dent Sci.

[6] Zhang X, Zeng D, Huang F, Wang J (2019). A protocol for isolation and culture of mesenchymal stem cells from human gingival tissue. Am J Clin Exp Immunol.

[7] Li J, Xu SQ, Zhao YM, Yu S, Ge LH, Xu BH (2018). Comparison of the biological characteristics of human mesenchymal stem cells derived from exfoliated deciduous teeth, bone marrow, gingival tissue, and umbilical cord. Mol Med Report.

[8] Chen X, Chen Y, Hou Y, Song P, Zhou M, Nie M (2019). Modulation of proliferation and differentiation of gingiva-derived mesenchymal stem cells by concentrated growth factors: potential implications in tissue engineering for dental regeneration and repair. Int J Mol Med.

[9] Yuan W-X, Wang X-X, Zheng D-H, Ma D, Cui Q, Yang F (2019). Muscone promotes the adipogenic differentiation of human gingival mesenchymal stem cells by inhibiting the Wnt/β-catenin signaling pathway. Drug Des Devel Ther.

[10] Subbarayan R, Murugan Girija D, Ranga RS (2018). Gingival spheroids possess multilineage differentiation potential. J Cell Physiol.

[11] Li D, Zou X-Y, El-Ayachi I, Romero LO, Yu Z, Iglesias-Linares A (2019). Human dental pulp stem cells and gingival mesenchymal stem cells display action potential capacity in vitro after neuronogenic differentiation. Stem Cell Rev.

[12] Buranasin P, Mizutani K, Iwasaki K, Pawaputanon N, Mahasarakham C, Kido D, Takeda K (2018). High glucose-induced oxidative stress impairs proliferation and migration of human gingival fibroblasts. PLoS ONE.

[13] Sahu M, Suryawanshi H, Nayak S, Kumar P (2017). Cytomorphometric analysis of gingival epithelium and buccal mucosa cells in type 2 diabetes mellitus patients. J Oral Maxillofacial Pathol JOMFP.

[14] Zhang P, Lu B, Zhu R, Yang D, Liu W, Wang Q (2021). Hyperglycemia accelerates inflammaging in the gingival epithelium through inflammasomes activation. J Periodontal Res.

[15] Kido D, Mizutani K, Takeda K, Mikami R, Matsuura T, Iwasaki K (2017). Impact of diabetes on gingival wound healing via oxidative stress. PLoS ONE.

[16] Chainy GB, Sahoo DK (2020). Hormones and oxidative stress: an overview. Free Radical Res.

[17] Carneiro BA, El-Deiry WS (2020). Targeting apoptosis in cancer therapy. Nat Rev Clin Oncol.

[18] D’Arcy MS (2019). Cell death: a review of the major forms of apoptosis, necrosis and autophagy. Cell Biol Int.

[19] Abate M, Festa A, Falco M, Lombardi A, Luce A, Grimaldi A, et al., editors. Mitochondria as playmakers of apoptosis, autophagy and senescence. Seminars in cell & developmental biology; 2020: Elsevier. 10.1016/j.semcdb.2019.05.022 31154010

[20] Voss AK, Strasser A. The essentials of developmental apoptosis. F1000Research. 2020;9. 10.12688/f1000research.21571.1 PMC7047912 32148779

[21] Rawal I, Ghosh S, Hameed SS, Shivashankar R, Ajay VS, Patel SA (2019). Association between poor oral health and diabetes among Indian adult population: potential for integration with NCDs. BMC Oral Health.

[22] Jin SH, Lee J, Yun JH, Kim I, Ko Y, Park J-B (2015). Isolation and characterization of human mesenchymal stem cells from gingival connective tissue. J Periodontal Res.

[23] Gugliandolo A, Diomede F, Cardelli P, Bramanti A, Scionti D, Bramanti P (2018). Transcriptomic analysis of gingival mesenchymal stem cells cultured on 3 d bioprinted scaffold: a promising strategy for neuroregeneration. J Biomed Mater Res Part A.

[24] Li N, Liu N, Zhou J, Tang L, Ding B, Duan Y (2013). Inflammatory environment induces gingival tissue-specific mesenchymal stem cells to differentiate towards a pro-fibrotic phenotype. Biol Cell.

[25] Zhang Y, Xing Y, Jia L, Ji Y, Zhao B, Wen Y (2018). An in vitro comparative study of multisource derived human mesenchymal stem cells for bone tissue engineering. Stem Cells Dev.

[26] Wang W, Zhao H, Chen B (2020). DJ-1 protects retinal pericytes against high glucose-induced oxidative stress through the Nrf2 signaling pathway. Sci Rep.

[27] Fujimoto K, Ford EL, Tran H, Wice BM, Crosby SD, Dorn GW (2010). Loss of Nix in Pdx1-deficient mice prevents apoptotic and necrotic β cell death and diabetes. J Clin Investig.

[28] Nie Z, Chen S, Deng S, Long L, Peng P, Gao M (2018). Gene expression profiling of osteoblasts subjected to dexamethasone-induced apoptosis with/without GSK3β-shRNA. Biochem Biophys Res Commun.

[29] Janus P, Toma-Jonik A, Vydra N, Mrowiec K, Korfanty J, Chadalski M (2020). Pro-death signaling of cytoprotective heat shock factor 1: upregulation of NOXA leading to apoptosis in heat-sensitive cells. Cell Death Differ.

[30] Al-Serwi RH, Othman G, Attia MA, Enan ET, El-Sherbiny M, Mahmoud S (2020). Enhancement of cisplatin cytotoxicity by Cu (II)–Mn (II) schiff base tetradentate complex in human oral squamous cell carcinoma. Molecules.

[31] Zhang XM, Wang YZ, Tong JD, Ning XC, Zhou FQ, Yang XH (2020). Pyruvate alleviates high glucose-induced endoplasmic reticulum stress and apoptosis in HK-2 cells. FEBS Open Bio.

[32] Chen M-F, Liou S-S, Hong T-Y, Kao S-T, Liu I-M (2019). Gigantol has protective effects against high glucose-evoked nephrotoxicity in mouse glomerulus mesangial cells by suppressing ROS/MAPK/NF-κB signaling pathways. Molecules.

[33] Honma K, Machida C, Mochizuki K, Goda T (2020). Glucose and TNF enhance expression of TNF and IL1B, and histone H3 acetylation and K4/K36 methylation, in juvenile macrophage cells. Gene X.

[34] Luo H, Zhu W, Mo W, Liang M (2020). High-glucose concentration aggravates TNF-alpha-induced cell viability reduction in human CD146-positive periodontal ligament cells via TNFR-1 gene demethylation. Cell Biol Int.

[35] Zhu W, Qiu Q, Luo H, Zhang F, Wu J, Zhu X, Liang M (2020). High glucose exacerbates TNF-α-induced proliferative inhibition in human periodontal ligament stem cells through upregulation and activation of TNF receptor 1. Stem Cells Int.

[36] Xiong W, Wu Y, Xian W, Song L, Hu L, Pan S (2018). DAPK1-ERK signal mediates oxygen glucose deprivation reperfusion induced apoptosis in mouse N2a cells. J Neurol Sci.

[37] Geng J, Ito Y, Shi L, Amin P, Chu J, Ouchida AT (2017). Regulation of RIPK1 activation by TAK1-mediated phosphorylation dictates apoptosis and necroptosis. Nat Commun.

[38] Panahi G, Pasalar P, Zare M, Rizzuto R, Meshkani R (2018). High glucose induces inflammatory responses in HepG2 cells via the oxidative stress-mediated activation of NF-κB, and MAPK pathways in HepG2 cells. Arch Physiol Biochem.

[39] Oróstica L, García P, Vera C, García V, Romero C, Vega M (2018). Effect of TNF-α on molecules related to the insulin action in endometrial cells exposed to hyperandrogenic and hyperinsulinic conditions characteristics of polycystic ovary syndrome. Reprod Sci.

[40] Alnahdi A, John A, Raza H (2019). Augmentation of glucotoxicity, oxidative stress, apoptosis and mitochondrial dysfunction in HepG2 cells by palmitic acid. Nutrients.

