# Supplementary material for: Effect of glucose mediated oxidative stress on apoptotic gene expression in gingival mesenchymal stem cells
Source: BMC Oral Health. 2021 Dec 18;21:653. doi: 10.1186/s12903-021-02007-y (PMC8684132; doi:10.1186/s12903-021-02007-y)
Supplement: Supplementary file 1 — Additional file 1. Table 1. CT values of housekeeping genes. Table 2. The values of average CT for apoptotic genes. Table 3. Normalization of data with 18s housekeeping gene. Table 4. Normalization of data with ACTB and GAPDH housekeeping gene. [file 12903_2021_2007_MOESM1_ESM.docx]

***Table 1:*** ***CT values of housekeeping genes***

| HOUSEKEEPING GENE | AVG. CT OF CONTROL | AVG. CT OF TREATED CELLS |
| --- | --- | --- |
| 18S | 7.33 | 7.26 |
| ACTB | 17.71 | 18.07 |
| GAPDH | 19.25 | 18.52 |

***Table 2: The values of average CT for apoptotic genes***

|  | **CONTROL** | **TREATED** |  | **CONTROL** | **TREATED** |
| --- | --- | --- | --- | --- | --- |
| **Target Name** | CT Average | CT Average | **Target Name** | CT Average | CT Average |
| APAF1 | 27.27 | 26.85 | BID | 27.32 | 27.31 |
| BAD | 26.53 | 27.09 | BIK | 40.00 | 40.00 |
| BAK1 | 27.98 | 28.03 | BIRC1 | 29.47 | 29.74 |
| BAX | 23.12 | 22.53 | BIRC2 | 26.30 | 25.20 |
| BBC3 | 26.93 | 26.98 | BIRC3 | 31.27 | 31.52 |
| BCAP31 | 23.09 | 23.06 | BIRC4 | 27.25 | 26.32 |
| BCL10 | 25.93 | 24.91 | BIRC5 | 26.92 | 28.34 |
| BCL2A1 | 35.40 | 33.07 | BIRC6 | 25.28 | 24.23 |
| BCL2 | 29.98 | 33.35 | BIRC7 | 40.00 | 40.00 |
| BCL2L10 | 40.00 | 40.00 | BIRC8 | 31.08 | 31.60 |
| BCL2L11 | 27.82 | 25.95 | BNIP3 | 24.25 | 23.45 |
| BCL2L13 | 25.10 | 24.42 | BNIP3L | 23.36 | 21.95 |
| BCL2L14 | 40.00 | 35.03 | BOK | 28.03 | 28.13 |
| BCL2L1 | 25.88 | 25.37 | CARD15 | 33.57 | 32.75 |
| BCL2L2 | 26.14 | 25.67 | CARD4 | 31.28 | 31.37 |
| 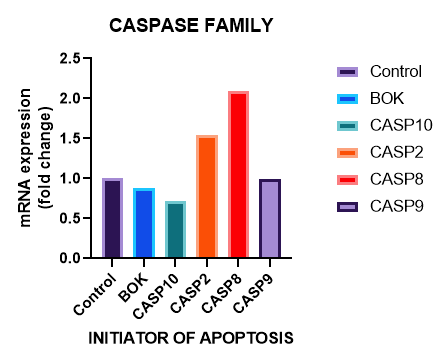BCL3 | 26.11 | 24.78 | CARD6 | 29.03 | 27.16 |
| CARD9 | 32.71 | 34.68 | PMAIP1 | 29.07 | 26.56 |
| CASP10 | 30.36 | 30.76 | PYCARD | 26.09 | 25.12 |
| CASP14 | 40.00 | 40.00 | RELA | 26.16 | 25.15 |
| CASP1 | 26.61 | 26.10 | RELB | 28.79 | 28.87 |
| CASP2 | 28.01 | 27.31 | REL | 30.12 | 28.88 |
| CASP3 | 27.22 | 26.21 | RIPK1 | 26.93 | 25.37 |
| CASP4 | 25.01 | 24.27 | RIPK2 | 26.93 | 25.87 |
| CASP5 | 35.52 | 34.99 | TA-NFKBH | 29.95 | 29.43 |
| CASP6 | 27.43 | 26.59 | TBK1 | 26.75 | 25.11 |
| CASP7 | 26.81 | 25.95 | TNF | 40.00 | 33.30 |
| CASP8AP2 | 28.50 | 27.39 | TNFRSF10A | 28.17 | 27.22 |
| CASP8 | 27.82 | 26.68 | TNFRSF10B | 24.88 | 24.61 |
| CASP9 | 26.27 | 26.21 | TNFRSF1A | 23.04 | 22.13 |
| CFLAR | 24.91 | 24.00 | TNFRSF1B | 28.52 | 27.55 |
| CHUK | 27.84 | 27.09 | TNFRSF21 | 27.21 | 27.99 |
| CRADD | 27.24 | 26.97 | TNFRSF25 | 31.81 | 31.98 |
| DAPK1 | 27.39 | 25.60 | TNFSF10 | 30.05 | 24.44 |
| DEDD2 | 28.08 | 27.71 | TRADD | 28.30 | 28.20 |
| DEDD | 26.09 | 24.70 |  |  |  |
| DIABLO | 25.79 | 25.25 |  |  |  |
| ESRRBL1 | 25.99 | 25.75 |  |  |  |
| FADD | 28.24 | 28.44 |  |  |  |
| FAS | 25.51 | 25.20 |  |  |  |
| FASLG | 40.00 | 35.72 |  |  |  |
| HIP1 | 25.99 | 25.26 |  |  |  |
| HRK | 34.16 | 34.85 |  |  |  |
| HTRA2 | 26.27 | 25.17 |  |  |  |
| ICEBERG | 35.28 | 40.00 |  |  |  |
| IKBKB | 27.30 | 26.71 |  |  |  |
| IKBKE | 29.07 | 27.57 |  |  |  |
| IKBKG | 27.09 | 26.74 |  |  |  |
| LRDD | 28.28 | 27.61 |  |  |  |
| LTA | 40.00 | 37.85 |  |  |  |
| LTB | 34.19 | 34.90 |  |  |  |
| MCL1 | 24.78 | 24.19 |  |  |  |
| NALP1 | 28.24 | 27.05 |  |  |  |
| NFKB1 | 26.57 | 25.54 |  |  |  |
| NFKB2 | 26.84 | 26.02 |  |  |  |
| NFKBIA | 25.69 | 23.40 |  |  |  |
| NFKBIB | 28.42 | 27.71 |  |  |  |
| NFKBIE | 30.69 | 30.12 |  |  |  |
| NFKBIZ | 26.59 | 27.00 |  |  |  |
| PEA15 | 21.49 | 20.81 |  |  |  |

***Table 3: Normalization of data with 18s housekeeping gene***

|  | **CONTROL** | **TREATED** | **TREATED-CONTROL** |  |  | **CONTROL** | **TREATED** | **TREATED-CONTROL** |  |
| --- | --- | --- | --- | --- | --- | --- | --- | --- | --- |
| **Target Name** | **∆CT 18s** | **∆CT 18s** | **∆∆CT** | **2^-∆∆CT** | **Target Name** | **∆CT 18s** | **∆CT 18s** | **∆∆CT** | **2^-∆∆CT** |
| APAF1 | 19.94 | 19.58 | -0.36 | 1.28 | CASP3 | 19.89 | 18.95 | -0.94 | 1.91 |
| BAD | 19.20 | 19.83 | 0.62 | 0.65 | CASP4 | 17.67 | 17.00 | -0.67 | 1.59 |
| BAK1 | 20.65 | 20.76 | 0.12 | 0.92 | CASP5 | 28.19 | 27.73 | -0.46 | 1.37 |
| BAX | 15.79 | 15.27 | -0.52 | 1.43 | CASP6 | 20.10 | 19.33 | -0.77 | 1.70 |
| BBC3 | 19.60 | 19.72 | 0.12 | 0.92 | CASP7 | 19.48 | 18.68 | -0.80 | 1.74 |
| BCAP31 | 15.76 | 15.79 | 0.04 | 0.97 | CASP8AP2 | 21.17 | 20.13 | -1.04 | 2.06 |
| BCL10 | 18.60 | 17.65 | -0.95 | 1.93 | CASP8 | 20.49 | 19.41 | -1.08 | 2.11 |
| BCL2A1 | 28.07 | 25.81 | -2.26 | 4.80 | CASP9 | 18.94 | 18.94 | 0.01 | 1.00 |
| BCL2 | 22.65 | 26.09 | 3.44 | 0.09 | CFLAR | 17.58 | 16.74 | -0.84 | 1.79 |
| BCL2L10 | 32.67 | 32.74 | 0.07 | 0.95 | CHUK | 20.50 | 19.83 | -0.68 | 1.60 |
| BCL2L11 | 20.49 | 18.69 | -1.80 | 3.49 | CRADD | 19.91 | 19.71 | -0.21 | 1.15 |
| BCL2L13 | 17.77 | 17.15 | -0.61 | 1.53 | DAPK1 | 20.05 | 18.34 | -1.72 | 3.29 |
| BCL2L14 | 32.67 | 27.77 | -4.90 | 29.80 | DEDD2 | 20.75 | 20.45 | -0.30 | 1.24 |
| BCL2L1 | 18.55 | 18.11 | -0.45 | 1.36 | DEDD | 18.76 | 17.44 | -1.32 | 2.50 |
| BCL2L2 | 18.81 | 18.41 | -0.40 | 1.32 | DIABLO | 18.46 | 17.98 | -0.47 | 1.39 |
| BCL3 | 18.78 | 17.52 | -1.27 | 2.40 | ESRRBL1 | 18.66 | 18.48 | -0.18 | 1.13 |
| BID | 19.99 | 20.05 | 0.06 | 0.96 | FADD | 20.91 | 21.18 | 0.27 | 0.83 |
| BIK | 32.67 | 32.74 | 0.07 | 0.95 | FAS | 18.18 | 17.94 | -0.25 | 1.19 |
| BIRC1 | 22.14 | 22.48 | 0.34 | 0.79 | FASLG | 32.67 | 28.46 | -4.21 | 18.53 |
| BIRC2 | 18.97 | 17.93 | -1.04 | 2.05 | HIP1 | 18.66 | 17.99 | -0.67 | 1.59 |
| BIRC3 | 23.94 | 24.26 | 0.32 | 0.80 | HRK | 26.83 | 27.59 | 0.76 | 0.59 |
| BIRC4 | 19.92 | 19.05 | -0.87 | 1.82 | HTRA2 | 18.94 | 17.91 | -1.03 | 2.04 |
| BIRC5 | 19.59 | 21.08 | 1.49 | 0.36 | ICEBERG | 27.95 | 32.74 | 4.79 | 0.04 |
| BIRC6 | 17.95 | 16.96 | -0.99 | 1.98 | IKBKB | 19.97 | 19.45 | -0.52 | 1.43 |
| BIRC7 | 32.67 | 32.74 | 0.07 | 0.95 | IKBKE | 21.74 | 20.31 | -1.43 | 2.70 |
| BIRC8 | 23.75 | 24.34 | 0.58 | 0.67 | IKBKG | 19.76 | 19.48 | -0.28 | 1.21 |
| BNIP3 | 16.92 | 16.19 | -0.73 | 1.66 | LRDD | 20.95 | 20.35 | -0.60 | 1.51 |
| BNIP3L | 16.03 | 14.69 | -1.34 | 2.53 | LTA | 32.67 | 30.59 | -2.08 | 4.23 |
| BOK | 20.70 | 20.86 | 0.16 | 0.89 | LTB | 26.85 | 27.64 | 0.78 | 0.58 |
| CARD15 | 26.24 | 25.49 | -0.75 | 1.68 | MCL1 | 17.45 | 16.93 | -0.52 | 1.43 |
| CARD4 | 23.95 | 24.11 | 0.16 | 0.90 | NALP1 | 20.91 | 19.79 | -1.12 | 2.18 |
| CARD6 | 21.70 | 19.90 | -1.80 | 3.48 | NFKB1 | 19.23 | 18.28 | -0.95 | 1.94 |
| CARD9 | 25.38 | 27.42 | 2.03 | 0.24 | NFKB2 | 19.51 | 18.76 | -0.76 | 1.69 |
| CASP10 | 23.03 | 23.50 | 0.47 | 0.72 | NFKBIA | 18.36 | 16.14 | -2.23 | 4.68 |
| CASP14 | 32.67 | 32.74 | 0.07 | 0.95 | NFKBIB | 21.09 | 20.45 | -0.64 | 1.56 |
| CASP1 | 19.28 | 18.84 | -0.44 | 1.36 | NFKBIE | 23.35 | 22.86 | -0.50 | 1.41 |
| CASP2 | 20.68 | 20.04 | -0.64 | 1.56 | NFKBIZ | 19.26 | 19.74 | 0.48 | 0.72 |
| PEA15 | 14.15 | 13.55 | -0.61 | 1.53 |  |  |  |  |  |
| PMAIP1 | 21.74 | 19.29 | -2.44 | 5.43 |  |  |  |  |  |
| PYCARD | 18.76 | 17.86 | -0.90 | 1.87 |  |  |  |  |  |
| RELA | 18.83 | 17.89 | -0.94 | 1.91 |  |  |  |  |  |
| RELB | 21.46 | 21.60 | 0.14 | 0.91 |  |  |  |  |  |
| REL | 22.78 | 21.62 | -1.17 | 2.24 |  |  |  |  |  |
| RIPK1 | 19.60 | 18.11 | -1.49 | 2.81 |  |  |  |  |  |
| RIPK2 | 19.60 | 18.60 | -0.99 | 1.99 |  |  |  |  |  |
| TANFKBH | 22.62 | 22.16 | -0.46 | 1.37 |  |  |  |  |  |
| TBK1 | 19.42 | 17.85 | -1.57 | 2.97 |  |  |  |  |  |
| TNF | 32.67 | 26.04 | -6.63 | 99.15 |  |  |  |  |  |
| TNFRSF10A | 20.84 | 19.96 | -0.88 | 1.84 |  |  |  |  |  |
| TNFRSF10B | 17.55 | 17.35 | -0.21 | 1.15 |  |  |  |  |  |
| TNFRSF1A | 15.71 | 14.87 | -0.84 | 1.79 |  |  |  |  |  |
| TNFRSF1B | 21.19 | 20.29 | -0.91 | 1.87 |  |  |  |  |  |
| TNFRSF21 | 19.88 | 20.73 | 0.85 | 0.56 |  |  |  |  |  |
| TNFRSF25 | 24.48 | 24.71 | 0.23 | 0.85 |  |  |  |  |  |
| TNFSF10 | 22.72 | 17.17 | -5.55 | 46.77 |  |  |  |  |  |
| TRADD | 20.97 | 20.93 | -0.03 | 1.02 |  |  |  |  |  |

***Table 4: Normalization of data with ACTB and GAPDH housekeeping gene***

|  | **CONTROL** | **TREATED** |  |  | **CONTROL** | **TREATED** |  |  |
| --- | --- | --- | --- | --- | --- | --- | --- | --- |
| **Target Name** | **∆CT ACTB** | **∆CT ACTB** | **∆∆CT** | **2^-∆∆CT** | **∆CT GAPDH** | **∆CT GAPDH** | **∆∆CT** | **2^-∆∆CT** |
| APAF1 | 9.56 | 8.78 | -0.78 | 1.72 | 8.02 | 8.32 | 0.30 | 0.81 |
| BAD | 8.83 | 9.02 | 0.19 | 0.87 | 7.29 | 8.57 | 1.28 | 0.41 |
| BAK1 | 10.27 | 9.96 | -0.31 | 1.24 | 8.73 | 9.50 | 0.78 | 0.58 |
| BAX | 5.41 | 4.47 | -0.94 | 1.92 | 3.87 | 4.01 | 0.14 | 0.90 |
| BBC3 | 9.23 | 8.92 | -0.31 | 1.24 | 7.68 | 8.46 | 0.78 | 0.58 |
| BCAP31 | 5.38 | 4.99 | -0.39 | 1.31 | 3.84 | 4.54 | 0.70 | 0.62 |
| BCL10 | 8.22 | 6.85 | -1.37 | 2.59 | 6.68 | 6.39 | -0.29 | 1.22 |
| BCL2A1 | 17.69 | 15.00 | -2.69 | 6.46 | 16.15 | 14.55 | -1.60 | 3.04 |
| BCL2 | 12.28 | 15.28 | 3.01 | 0.12 | 10.73 | 14.83 | 4.10 | 0.06 |
| BCL2L10 | 22.29 | 21.93 | -0.36 | 1.28 | 20.75 | 21.48 | 0.73 | 0.60 |
| BCL2L11 | 10.12 | 7.88 | -2.23 | 4.70 | 8.57 | 7.43 | -1.14 | 2.21 |
| BCL2L13 | 7.39 | 6.35 | -1.04 | 2.06 | 5.85 | 5.90 | 0.05 | 0.97 |
| BCL2L14 | 22.29 | 16.97 | -5.33 | 40.13 | 20.75 | 16.51 | -4.24 | 18.86 |
| BCL2L1 | 8.18 | 7.30 | -0.87 | 1.83 | 6.63 | 6.85 | 0.21 | 0.86 |
| BCL2L2 | 8.44 | 7.61 | -0.83 | 1.78 | 6.89 | 7.15 | 0.26 | 0.83 |
| BCL3 | 8.41 | 6.71 | -1.69 | 3.24 | 6.86 | 6.26 | -0.61 | 1.52 |
| BID | 9.61 | 9.25 | -0.37 | 1.29 | 8.07 | 8.79 | 0.72 | 0.61 |
| BIK | 22.29 | 21.93 | -0.36 | 1.28 | 20.75 | 21.48 | 0.73 | 0.60 |
| BIRC1 | 11.76 | 11.67 | -0.09 | 1.06 | 10.22 | 11.22 | 1.00 | 0.50 |
| BIRC2 | 8.60 | 7.13 | -1.47 | 2.76 | 7.05 | 6.68 | -0.38 | 1.30 |
| BIRC3 | 13.56 | 13.46 | -0.11 | 1.08 | 12.02 | 13.00 | 0.98 | 0.51 |
| BIRC4 | 9.55 | 8.25 | -1.30 | 2.46 | 8.00 | 7.80 | -0.21 | 1.15 |
| BIRC5 | 9.22 | 10.27 | 1.06 | 0.48 | 7.67 | 9.82 | 2.15 | 0.23 |
| BIRC6 | 7.58 | 6.16 | -1.42 | 2.67 | 6.03 | 5.71 | -0.33 | 1.25 |
| BIRC7 | 22.29 | 21.93 | -0.36 | 1.28 | 20.75 | 21.48 | 0.73 | 0.60 |
| BIRC8 | 13.38 | 13.53 | 0.16 | 0.90 | 11.83 | 13.08 | 1.24 | 0.42 |
| BNIP3 | 6.55 | 5.38 | -1.16 | 2.24 | 5.00 | 4.93 | -0.07 | 1.05 |
| BNIP3L | 5.66 | 3.89 | -1.77 | 3.41 | 4.11 | 3.43 | -0.68 | 1.60 |
| BOK | 10.32 | 10.06 | -0.26 | 1.20 | 8.78 | 9.60 | 0.82 | 0.56 |
| CARD15 | 15.87 | 14.69 | -1.18 | 2.27 | 14.32 | 14.23 | -0.09 | 1.07 |
| CARD4 | 13.58 | 13.31 | -0.27 | 1.21 | 12.03 | 12.85 | 0.82 | 0.57 |
| CARD6 | 11.33 | 9.10 | -2.23 | 4.69 | 9.78 | 8.64 | -1.14 | 2.21 |
| CARD9 | 15.01 | 16.61 | 1.60 | 0.33 | 13.46 | 16.16 | 2.69 | 0.15 |
| CASP10 | 12.65 | 12.70 | 0.04 | 0.97 | 11.11 | 12.24 | 1.13 | 0.46 |
| CASP14 | 22.29 | 21.93 | -0.36 | 1.28 | 20.75 | 21.48 | 0.73 | 0.60 |
| CASP1 | 8.91 | 8.03 | -0.87 | 1.83 | 7.36 | 7.58 | 0.22 | 0.86 |
| CASP2 | 10.31 | 9.24 | -1.07 | 2.10 | 8.76 | 8.78 | 0.02 | 0.99 |
| CASP3 | 9.51 | 8.15 | -1.36 | 2.58 | 7.97 | 7.69 | -0.28 | 1.21 |
| CASP4 | 7.30 | 6.20 | -1.10 | 2.14 | 5.76 | 5.75 | -0.01 | 1.01 |
| CASP5 | 17.81 | 16.93 | -0.89 | 1.85 | 16.27 | 16.47 | 0.20 | 0.87 |
| CASP6 | 9.72 | 8.53 | -1.20 | 2.29 | 8.18 | 8.07 | -0.11 | 1.08 |
| CASP7 | 9.11 | 7.88 | -1.23 | 2.34 | 7.56 | 7.43 | -0.14 | 1.10 |
| CASP8AP2 | 10.79 | 9.32 | -1.47 | 2.77 | 9.25 | 8.87 | -0.38 | 1.30 |
| CASP8 | 10.12 | 8.61 | -1.51 | 2.84 | 8.57 | 8.15 | -0.42 | 1.34 |
| CASP9 | 8.56 | 8.14 | -0.42 | 1.34 | 7.02 | 7.69 | 0.67 | 0.63 |
| CFLAR | 7.20 | 5.94 | -1.27 | 2.40 | 5.66 | 5.48 | -0.18 | 1.13 |
| CHUK | 10.13 | 9.02 | -1.11 | 2.16 | 8.59 | 8.57 | -0.02 | 1.01 |
| CRADD | 9.54 | 8.90 | -0.64 | 1.55 | 7.99 | 8.45 | 0.45 | 0.73 |
| DAPK1 | 9.68 | 7.53 | -2.15 | 4.43 | 8.14 | 7.08 | -1.06 | 2.08 |
| DEDD2 | 10.38 | 9.64 | -0.73 | 1.66 | 8.83 | 9.19 | 0.36 | 0.78 |
| DEDD | 8.39 | 6.64 | -1.75 | 3.36 | 6.84 | 6.18 | -0.66 | 1.58 |
| DIABLO | 8.09 | 7.18 | -0.90 | 1.87 | 6.54 | 6.73 | 0.19 | 0.88 |
| ESRRBL1 | 8.29 | 7.68 | -0.61 | 1.52 | 6.74 | 7.22 | 0.48 | 0.72 |
| FADD | 10.54 | 10.38 | -0.16 | 1.12 | 8.99 | 9.92 | 0.93 | 0.52 |
| FAS | 7.81 | 7.13 | -0.68 | 1.60 | 6.27 | 6.68 | 0.41 | 0.75 |
| FASLG | 22.29 | 17.65 | -4.64 | 24.95 | 20.75 | 17.20 | -3.55 | 11.73 |
| HIP1 | 8.29 | 7.19 | -1.10 | 2.14 | 6.74 | 6.74 | -0.01 | 1.01 |
| HRK | 16.46 | 16.78 | 0.33 | 0.80 | 14.91 | 16.33 | 1.42 | 0.37 |
| HTRA2 | 8.56 | 7.11 | -1.46 | 2.75 | 7.02 | 6.65 | -0.37 | 1.29 |
| ICEBERG | 17.57 | 21.93 | 4.36 | 0.05 | 16.03 | 21.48 | 5.45 | 0.02 |
| IKBKB | 9.59 | 8.65 | -0.95 | 1.93 | 8.05 | 8.19 | 0.14 | 0.91 |
| IKBKE | 11.36 | 9.50 | -1.86 | 3.63 | 9.82 | 9.05 | -0.77 | 1.71 |
| IKBKG | 9.38 | 8.68 | -0.71 | 1.63 | 7.84 | 8.22 | 0.38 | 0.77 |
| LRDD | 10.57 | 9.54 | -1.03 | 2.04 | 9.03 | 9.09 | 0.06 | 0.96 |
| LTA | 22.29 | 19.79 | -2.51 | 5.69 | 20.75 | 19.33 | -1.42 | 2.67 |
| LTB | 16.48 | 16.83 | 0.35 | 0.78 | 14.94 | 16.38 | 1.44 | 0.37 |
| MCL1 | 7.07 | 6.13 | -0.95 | 1.93 | 5.53 | 5.67 | 0.14 | 0.91 |
| NALP1 | 10.54 | 8.99 | -1.55 | 2.93 | 8.99 | 8.53 | -0.46 | 1.38 |
| NFKB1 | 8.86 | 7.48 | -1.38 | 2.61 | 7.32 | 7.02 | -0.29 | 1.23 |
| NFKB2 | 9.14 | 7.95 | -1.18 | 2.27 | 7.59 | 7.50 | -0.10 | 1.07 |
| NFKBIA | 7.99 | 5.33 | -2.65 | 6.30 | 6.44 | 4.88 | -1.57 | 2.96 |
| NFKBIB | 10.71 | 9.65 | -1.07 | 2.09 | 9.17 | 9.19 | 0.02 | 0.98 |
| NFKBIE | 12.98 | 12.05 | -0.93 | 1.90 | 11.44 | 11.60 | 0.16 | 0.89 |
| NFKBIZ | 8.89 | 8.94 | 0.05 | 0.97 | 7.34 | 8.48 | 1.14 | 0.45 |
| PEA15 | 3.78 | 2.74 | -1.04 | 2.05 | 2.24 | 2.29 | 0.05 | 0.97 |
| PMAIP1 | 11.36 | 8.49 | -2.87 | 7.31 | 9.82 | 8.04 | -1.78 | 3.44 |
| PYCARD | 8.38 | 7.05 | -1.33 | 2.51 | 6.84 | 6.60 | -0.24 | 1.18 |
| RELA | 8.45 | 7.09 | -1.37 | 2.58 | 6.91 | 6.63 | -0.28 | 1.21 |
| RELB | 11.09 | 10.80 | -0.29 | 1.22 | 9.55 | 10.35 | 0.80 | 0.57 |
| REL | 12.41 | 10.82 | -1.59 | 3.02 | 10.87 | 10.36 | -0.51 | 1.42 |
| RIPK1 | 9.22 | 7.30 | -1.92 | 3.78 | 7.68 | 6.85 | -0.83 | 1.78 |
| RIPK2 | 9.22 | 7.80 | -1.42 | 2.68 | 7.68 | 7.34 | -0.33 | 1.26 |
| TA-NFKBH | 12.25 | 11.36 | -0.88 | 1.85 | 10.70 | 10.91 | 0.20 | 0.87 |
| TBK1 | 9.05 | 7.05 | -2.00 | 3.99 | 7.50 | 6.59 | -0.91 | 1.88 |
| TNF | 22.29 | 15.23 | -7.06 | 133.52 | 20.75 | 14.78 | -5.97 | 62.75 |
| TNFRSF10A | 10.47 | 9.16 | -1.31 | 2.48 | 8.92 | 8.70 | -0.22 | 1.17 |
| TNFRSF10B | 7.18 | 6.54 | -0.64 | 1.55 | 5.63 | 6.09 | 0.45 | 0.73 |
| TNFRSF1A | 5.34 | 4.06 | -1.27 | 2.41 | 3.79 | 3.61 | -0.18 | 1.13 |
| TNFRSF1B | 10.82 | 9.48 | -1.33 | 2.52 | 9.27 | 9.03 | -0.25 | 1.19 |
| TNFRSF21 | 9.50 | 9.92 | 0.42 | 0.75 | 7.96 | 9.47 | 1.51 | 0.35 |
| TNFRSF25 | 14.11 | 13.91 | -0.20 | 1.15 | 12.56 | 13.46 | 0.89 | 0.54 |
| TNFSF10 | 12.35 | 6.37 | -5.98 | 62.98 | 10.80 | 5.92 | -4.89 | 29.60 |
| TRADD | 10.59 | 10.13 | -0.46 | 1.38 | 9.05 | 9.68 | 0.63 | 0.65 |
